# Picking the Right Patient for Human Epidermal Growth Factor Receptor 3–Targeted Therapy in Platinum-Resistant Ovarian Cancer

**DOI:** 10.1200/JCO.2016.69.7169

**Published:** 2016-10-26

**Authors:** Alison M. Schram, Alexia Iasonos, David M. Hyman

**Affiliations:** Alison M. Schram, Alexia Iasonos, and David M. Hyman, Memorial Sloan Kettering Cancer Center, New York, NY

Surgical cytoreduction followed by platinum-based chemotherapy has been the standard first-line treatment of patients with high-risk early-stage and advanced epithelial ovarian cancer for nearly two decades.^1-4^ Although the majority of women with advanced disease will respond to combined platinum/taxane therapy, most will ultimately experience recurrence and eventually die as a result of their ovarian cancer. Platinum-refractory and -resistant disease is defined as progression on first-line chemotherapy or within 6 months of platinum completion, respectively. These patients are treated with nonplatinum chemotherapy; however, anticipated response rates are low.^5^ Despite the urgent need for more effective treatments, few new agents have demonstrated sufficient efficacy to warrant approval by the Food and Drug Administration (FDA) in the last 10 years. In 2014, the Avastin Use in Platinum-Resistant Epithelial Ovarian Cancer (AURELIA) trial established that the addition of bevacizumab to nonplatinum chemotherapy increases progression-free survival (PFS) from 3.4 to 6.7 months, leading to FDA approval.^6^ In 2015, Kaufman et al^7^ showed that olaparib achieved a 31% response rate in heavily pretreated women with germline *BRCA1/2* mutations, also leading to FDA approval in germline *BRCA1/2* carriers. Despite this recent progress, there remains a significant unmet need for improved therapies in platinum-resistant and -refractory ovarian cancer.

In the article that accompanies this editorial, Liu et al^8^ report the results of a 233-patient open-label, randomized phase II trial of once-weekly paclitaxel with or without the human epidermal growth factor receptor 3 (HER3) antibody, seribantumab, in platinum-resistant/refractory epithelial ovarian cancer. Patients were randomly assigned 2:1 in favor of seribantumab and enrolled without prospective biomarker selection or stratification. Unfortunately, the study did not reach its primary end point, showing no difference in PFS between the two arms (3.8 months with the combination compared with 3.7 months with paclitaxel alone). Despite this disappointing result, the study fortunately mandated the collection of both archival and fresh tumor biopsies, and these were used to conduct an extensive retrospective biomarker analysis to determine if a subset of patients may have benefited from the addition of seribantumab. By evaluating multiple biomarkers relevant to the mechanism of action of seribantumab, Liu et al^8^ identified a tumor-based bivariate signature defined by high heregulin (HRG, also named neuregulin 1), the ligand of HER3, and low human epidermal growth factor receptor 2 (HER2) that was predictive of seribantumab benefit. Specifically, in the 38% of evaluable patients who were biomarker positive, the median PFS was 5.7 months with the combination compared with 3.5 months with paclitaxel alone. In addition to being predictive of benefit to seribantumab, this signature seemed to be prognostic in the paclitaxel monotherapy arm, with biomarker-positive patients experiencing more rapid disease progression (PFS, 3.5 months *v* 5.4 months). Together, these observations suggest that seribantumab overcomes the negative prognosis associated with high HRG and low HER2 levels in platinum-resistant/refractory ovarian cancer. Distressingly, the biomarker-negative patients did worse when exposed to seribantumab, although the mechanism underlying this apparent harm is not understood.

HER3, the protein encoded by *ErbB3*, is a member of the human epidermal growth factor (EGFR) family and the only one that lacks catalytic kinase function. Instead, HER3 mediates its effects on signaling through heterodimerization with, and allosteric activation of, other EGFR family members, leading to downstream activation of the phosphatidylinositol 3-kinase/AKT pathway.^9-11^ Pertuzumab, an anti-HER2 monoclonal antibody, is believed to act by preventing dimerization with HER3 and has been approved for treatment of HER2-positive breast cancer, further credentialing HER3 as a therapeutic target in cancer.^12^ In ovarian cancer, HER3 is highly expressed in a subset of patients and is associated with a worse prognosis.^13^ Autocrine signaling loops between HER3 and its ligand, HRG, promote growth in patient-derived ovarian cancer models and cell lines.^14^ Treatment of ovarian cancer cell lines with certain chemotherapies increases activation of HER3, suggesting HER3 may be one of several mechanisms responsible for chemotherapy resistance in ovarian cancer.^15^ Seribantumab is a fully humanized monoclonal antibody that blocks binding of HRG to HER3 and has been shown to cause tumor growth arrest in ovarian cancer xenograph models.^16^ In tumors with high HRG expression, seribantumab is believed to block ligand-dependent activation of HER2/HER3 dimers. Conversely, high levels of HER2, leading to a greater presence of HER2/HER3 dimers, may mitigate therapeutic benefit by promoting ligand-independent signaling. Thus, the findings by Liu et al^8^ that the combination of high HRG and low HER2 levels were associated with benefit to seribantumab is consistent with preclinical predictions. In distinction, the finding that biomarker-negative patients fared worse when treated with seribantumab is not explained by this proposed mechanism and therefore raises important unanswered questions. Despite this, preliminary reports from studies in non–small-cell lung cancer and breast cancer have provided additional clinical corroboration of this predictive biomarker.^17,18^ There is currently an ongoing, potentially registration-enabling randomized phase II trial of chemotherapy with or without seribantumab in HRG-positive non–small-cell lung cancer.^19^

Although both the general association with and directionality of HRG and HER2 levels with benefit of seribantumab match preclinical expectations, some caution is appropriate when interpreting the results of the current study. Tumor levels of HRG, HER3, HER2, EGFR, and betacellulin (an EGF family ligand) were measured using four orthogonal techniques (reverse transcriptase quantitative polymerase chain reaction, RNA–in situ hybridization, fluorescence-based quantitative immunohistochemistry, and chromogenic RNA-in situ hybridization). In the resulting analysis, 13 unique marker/measurement combinations were tested for association with treatment outcome in both archived and fresh tumor biopsies. Because of the exploratory nature of the analysis, no modifications were used to account for multiple hypothesis testing, and therefore the possibility of false discovery cannot be excluded. On univariate analysis, all four analytes were significantly associated with outcome, although associations were not concordant across the different measurement techniques used for each analyte. Although this finding may be caused by differences in the performance of various quantification techniques when using limited quantity or degraded tumor material, the observation provides some cause for concern. Moreover, although the markers evaluated were prespecified by the protocol, the specifics of this analysis itself and cut points used were not. Finally, only 57 patients meeting criteria for biomarker positivity were enrolled in the current study, limiting our ability to estimate the true effect size of seribantumab in this population with precision.

Given these various considerations, our next steps as a field should be guided by how confident we are that this analysis has identified the optimal patient selection strategy for HER3-targeted therapy in platinum-resistant ovarian cancer. A definitive phase III superiority study pursuing these preliminary findings would require approximately 250 biomarker-positive patients if targeting a hazard ratio of 0.65 for the combination versus paclitaxel alone, assuming the reported PFS difference and using a two-sided type I error of 5% with 90% power. The prospect that biomarker-negative patients may be harmed by seribantumab would necessitate prospective selection of only biomarker-positive patients for enrollment. Assuming a biomarker positivity rate of 40%, this study would require screening of more than 600 patients. Given the discordant biomarker status defined using archival and pretreatment tumor biopsies, patients may require fresh biopsies for screening. Thus, a definitive phase III study would be a major undertaking for both investigators and patients. Does the current rigorous but ultimately exploratory biomarker analysis support moving forward in this manner without further clinical and analytic validation of this selection strategy in ovarian cancer? A more conservative alternative approach could be to conduct a smaller follow-up study using prospective biomarker selection and incorporating less stringent statistical controls to provide additional support for these preliminary observations and refine end points for a future definitive study.

In conclusion, we congratulate Liu et al^8^ for not only conducting a well-designed trial but also having the foresight to collect the biospecimens necessary to conduct a rigorous biomarker evaluation. Their efforts have identified a potential path forward for this drug in ovarian cancer, salvaging what otherwise would have been a negative study. Moving forward, it is critical that we follow in the example of Liu et al^8^ and ensure that we have the opportunity to learn from our failures to ultimately improve the outcome for our patients.
